# Real-Time Built-In Self-Test of MEMS Gyroscope Based on Quadrature Error Signal

**DOI:** 10.3390/mi12091115

**Published:** 2021-09-16

**Authors:** Rui Feng, Jiong Wang, Wei Qiao, Fu Wang, Ming Zhou, Xinglian Shang, Lei Yu, Liuhui Zhou, Shuwen Guo

**Affiliations:** 1 East China Institute of Photo-Electron IC, Suzhou 215163, China; 15962107282@163.com (W.Q.); phu727@126.com (F.W.); zmlove08@163.com (M.Z.); glsxl1994@163.com (X.S.); deep_yu@163.com (L.Y.); zhousat@sina.com (L.Z.); 2 School of Mechanical Engineering, Nanjing University of Science and Technology, Nanjing 210094, China; wjiongz@mail.njust.edu.cn; 3 School of Electronic and Information Engineering, Soochow University, Suzhou 251000, China; 13862044796@163.com

**Keywords:** quadrature error, built-in self-test (BIST), MEMS gyroscope

## Abstract

In high-reliability applications, the health condition of the MEMS gyroscope needs to be known in real time to ensure that the system does not fail due to the wrong output signal. Because the MEMS gyroscope self-test based on the principle of electrostatic force cannot be performed during the working state. We propose that by monitoring the quadrature error signal of the MEMS gyroscope in real time, an online self-test of the MEMS gyroscope can be realized. The correlation between the gyroscope’s quadrature error amplitude signal and the gyroscope scale factor and bias was theoretically analyzed. Based on the sixteen-sided cobweb-like MEMS gyroscope, the real-time built-in self-test (BIST) method of the MEMS gyroscope based on the quadrature error signal was verified. By artificially setting the control signal of the gyroscope to zero, we imitated several scenarios where the gyroscope malfunctioned. Moreover, a mechanical impact table was used to impact the gyroscope. After a 6000 g shock, the gyroscope scale factor, bias, and quadrature error amplitude changed by −1.02%, −5.76%, and −3.74%, respectively, compared to before the impact. The gyroscope failed after a 10,000 g impact, and the quadrature error amplitude changed −99.82% compared to before the impact. The experimental results show that, when the amplitude of the quadrature error signal seriously deviates from the original value, it can be determined that the gyroscope output signal is invalid.

## 1. Introduction

The MEMS gyroscope is small, lightweight, and low in price, and is widely used in civil and military fields. However, strong shocks and vibrations will cause the scale factor and bias of the MEMS gyroscope to change [1]. Usually, the scale factor and bias of the gyroscope used in the inertial navigation system are the values calibrated when leaving the factory or before installation. When the gyroscope has a large-scale factor or bias change during use, the inertial navigation system will produce large measurement errors, which may eventually lead to system failure. Limited by actual usage conditions, most MEMS gyroscopes are difficult to remove and recalibrate after installation. Therefore, it is very important to know whether the output signal of the gyroscope is reliable during usage.

In order to ensure the high reliability of MEMS gyroscope output data in high-reliability applications, the researchers of MEMS gyroscopes improve the robustness of MEMS gyroscopes by enhancing the reliability design [1,2,3,4,5]. They also use fault detection methods to check the gyroscope’s state when the gyroscope is powered on or during its work [6,7,8,9]. In some high-reliability applications, a redundant design method can be used to combine multiple MEMS gyroscopes [10], but this will result in a significant increase in the SWaP (Size Weight and Power) of the system.

Driven by the demand for high-reliability applications, the BIST (built-in self-test) technology of the MEMS device has developed rapidly [11,12]. The BIST of MEMS accelerometers and MEMS pressure sensors are relatively mature [13,14,15,16,17]. A typical MEMS accelerometer BIST applies a known electrostatic force on the MEMS structure, and then measures the output signal to determine whether or not the device is working properly. The BIST of the MEMS gyroscope can also be realized by using similar principles [6,7,8], applying a known specific driving signal to the MEMS structure of the gyroscope. Thus, the gyroscope structure is stimulated to perform a specific movement, and the movement signal is detected by the circuit. According to the detected signal, it can be inferred whether or not the gyroscope is working normally.

However, the electrostatic excitation self-test requires that an electrostatic force be applied to the device structure, which makes it difficult for the device to complete the detection of the input at the same time as the electrostatic excitation self-test. Therefore, the self-test based on the electrostatic excitation method is mostly carried out after the device is powered on, or in the periodic interval of the working measurement. Compared with MEMS accelerometers, MEMS gyroscopes usually use high-vacuum packaging to improve the mechanical sensitivity. The high Q value of the gyroscope means that the electrostatic excitation self-test excitation signal needs a long decay time before the next measurement. Thus, it takes a long time for the gyroscope to self-test using the electrostatic excitation method. In high-dynamic application scenarios, it is difficult to meet the requirements of a high data refresh rate by using the electrostatic excitation self-test method.

To achieve real-time MEMS gyroscope self-test, a continuous self-test method was attempted [9]. Two test signals were injected into the quadrature cancellation loop to traverse the entire signal path. Ideally, the two test signals were not interacting with the Coriolis signal, thus the self-test and the angular rate detection can be performed at the same time. To the authors’ knowledge, this is the first method to achieve a real-time MEMS gyroscope self-test. However, in order to prevent the test signals coupling to the Coriolis signal, and therefore increasing offset drift and noise, the amplitudes and the frequencies of the two test signals need to be designed very carefully.

High dynamic application scenarios are often accompanied by high impact and strong vibration. High impact and strong vibration can easily cause a MEMS gyroscope to malfunction. This kind of malfunction is most likely due to the failure of the MEMS structure.

In order to ensure that the output of the MEMS gyroscope in high-reliability applications such as platform stability and inertial guidance is absolutely correct and reliable, we propose a simple way to achieve a real-time MEMS gyroscope self-test. By detecting the MEMS gyroscope’s quadrature error signal in real time and analyzing the change of the signal, the MEMS gyroscope online real-time health self-test can be achieved.

We introduce the mechanism and composition of the quadrature error signal of the MEMS gyroscope in the first section. The second part details how to use the quadrature error signal of the MEMS gyroscope to complete an online BIST. Then, we introduce the structure and circuit based on a sixteen-sided cobweb-like disk resonator gyroscope in the third and fourth section, respectively. The simulations are described in the fifth section. The sixth and seventh sections detail the MEMS gyroscope online BIST experiment and conclusion, respectively.

## 2. Principle of the Online BIST of the MEMS Gyroscope

### 2.1. The Quadrature Error Signal of the Gyroscope

According to the structural form, MEMS mechanical gyroscopes can be divided into torsion type, tuning fork type, frame type, spherical shell type, cylindrical type, ring type, etc. According to the principle of driving and detection, they can be divided into electrostatic type, electromagnetic type, piezoelectric type, piezoresistive type, etc.

Although the materials, structures, and detection principles of MEMS gyroscopes are diverse, their basic working principles are all based on the Coriolis effect. That is, the mass of the gyroscope vibrates back and forth in the driving axis. When there is an angular velocity along the input axis, the Coriolis force will force the mass to vibrate along the sensitive axis. By detecting the vibration amplitude on the sensitive axis, the input angular velocity can be calculated.

**Figure 1:**
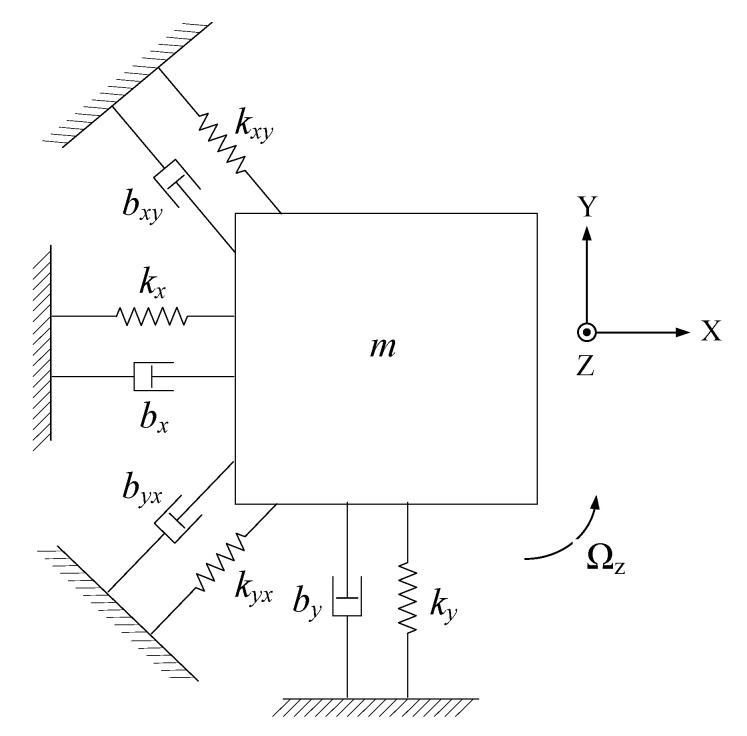
Schematic of the lumped parameter model of the MEMS gyroscope.

Due to inevitable fabrication errors, the structure of the MEMS gyroscope is not ideal. There is stiffness coupling and damping coupling between the gyroscope drive axis and the sense axis, as shown in Figure 1. The motion equation of the nonideal MEMS gyroscope structure can be expressed as [18]:

(1) $$\{\begin{array}{c} m_{x}\ddot{x}+b_{x}\dot{x}+b_{yx}\dot{y}+k_{x}x+k_{yx}y=F_{x}-2\dot{y}\Omega_{z}m_{x}\\ m_{y}\ddot{y}+b_{y}\dot{y}+b_{xy}\dot{x}+k_{y}y+k_{xy}x=F_{y}-2\dot{x}\Omega_{z}m_{y} \end{array}$$

where *x* and *y* are the displacement of the mass on the drive axis (X axis) and the sense axis (Y axis); *m_x_* and *m_y_* are the equivalent masses of the drive axis and the sense axis; *b_x_*, *b_y_*, *b_yx_* and *b_xy_* are the damping coefficients; *k_x_*, *k_y_*, *k_yx_* and *k_xy_* are the stiffness coefficients; Ω*_z_* is the input angular velocity of the Z axis; and *F_x_* and *F_y_* are the external forces of the drive axis and the sense axis.

Usually, the gyroscope sense modal displacement *y* is much smaller than the drive modal displacement *d*, so the Equation (1) is simplified to:

(2) $$\{\begin{array}{l} m_{x}\ddot{x}+b_{x}\dot{x}+k_{x}x=F_{x}\\ m_{y}\ddot{y}+b_{y}\dot{y}+k_{y}y=F_{y}-2\dot{x}\Omega_{z}m_{y}-b_{xy}\dot{x}-k_{xy}x \end{array}$$

Suppose the displacement of the drive axis is:

(3) $$x=A_{x}\cos\left(\omega_{d}t+\varphi_{x}\right)$$

where *A_x_* is the amplitude, *ω_d_* is the angular frequency of the drive signal, and *φ_x_* is the phase of the drive signal.

Combined Equations (2) and (3) gives:

(4) $$m_{y}\ddot{y}+b_{y}\dot{y}+k_{y}y=F_{y}+2\Omega_{z}m_{y}A_{x}\omega_{d}\sin\left(\omega_{d}t+\varphi_{x}\right)+b_{xy}A_{x}\omega_{d}\sin\left(\omega_{d}t+\varphi_{x}\right)-k_{xy}A_{x}\cos\left(\omega_{d}t+\varphi_{x}\right)$$

The in-phase feedback force and the quadrature feedback force of the MEMS gyroscope in the force balance working mode can be expressed as:

(5) $$\{\begin{array}{c} F_{I}=K_{s}V_{\Omega }\cos\left(\omega_{d}t+\varphi_{x}+\varphi_{s}\right)\\ F_{q}=K_{s}V_{q}\sin\left(\omega_{d}t+\varphi_{x}+\varphi_{s}\right) \end{array}$$

where *K_s_* is the amplification factor from feedback voltage to feedback force; *V*_Ω_ and *V_q_* are the in-phase feedback voltage signal (the amplitude of the Coriolis signal output by the circuit) and the quadrature feedback voltage signal (the amplitude of the quadrature signal output by the circuit), respectively; and *φ_s_* is the phase difference between the sensitive circuit and the drive circuit.

The sense modal displacement of the MEMS gyroscope in the force balance working mode is approximately zero. So:

(6) $$K_{s}V_{\Omega }\cos\left(\omega_{d}t+\varphi_{x}+\varphi_{s}\right)+K_{s}V_{q}\sin\left(\omega_{d}t+\varphi_{x}+\varphi_{s}\right)\;=2\Omega_{z}m_{y}A_{x}\omega_{d}\sin\left(\omega_{d}t+\varphi_{x}\right)+b_{xy}A_{x}\omega_{d}\sin\left(\omega_{d}t+\varphi_{x}\right)-k_{xy}A_{x}\cos\left(\omega_{d}t+\varphi_{x}\right)$$

Thus:

(7) $$\{\begin{array}{c} V_{q}=\frac{\left(2\Omega_{z}m_{y}A_{x}\omega_{d}+b_{xy}A_{x}\omega_{d}\right)\cos\left(\varphi_{s}\right)-k_{xy}A_{x}\sin\left(\varphi_{s}\right)}{K_{s}}\\ V_{\Omega }=\frac{-\left(2\Omega_{z}m_{y}A_{x}\omega_{d}+b_{xy}A_{x}\omega_{d}\right)\sin\left(\varphi_{s}\right)-k_{xy}A_{x}\cos\left(\varphi_{s}\right)}{K_{s}} \end{array}$$

Therefore, the bias of the gyroscope can be expressed as:

(8) $$V_{bias}=\frac{-b_{xy}A_{x}\omega_{d}\sin\left(\varphi_{s}\right)-k_{xy}A_{x}\cos\left(\varphi_{s}\right)}{K_{s}}$$

and the scale factor of the gyroscope can be expressed as:

(9) $$SF=\frac{-2m_{y}A_{x}\omega_{d}\sin\left(\varphi_{s}\right)}{K_{s}}$$

Since there is always a phase shift of the circuit, and the drive frequency of the gyroscope is not equal to the natural frequency of the drive modal, *φ_s_* is usually close, but not equal, to 90°. Thus, the actual output quadrature signal contains a weak angular velocity signal, and the actual output Coriolis signal contains a weak quadrature signal.

To reduce the quadrature error signal on the final output signal of the gyroscope, researchers have proposed many methods [19,20,21,22,23,24,25] to suppress the quadrature signal, and therefore to reduce the bias.

### 2.2. The BIST Based on Quadrature Error

Although the quadrature error signal needs to be minimized, the quadrature error signal is essentially a signal that reflects the motion state of the gyroscope structure. The quadrature error reflects the coupling of the drive mode vibration to the sense mode, so the quadrature error signal reflects both the vibration of the drive mode and the vibration of the sense mode. Meanwhile, the quadrature error signal is detected by the circuit of the gyroscope. It shares the same front-end detection circuit with the Coriolis signal, so the quadrature error signal also reflects the working status of the gyroscope sense modal front-end detection circuit.

From Equations (7)–(9), we can see that the bias of the gyro is related to the parameters *b_xy_*, *A_x_*, *ω_d_*, *k_xy_*, *φ_s_*, and *Ks*. The scale factor of the gyro is related to the parameters *m_y_*, *A_x_*, *ω_d_*, *φ_s_*, and *K_s_*. The quadrature error of the gyro is related to the parameters Ω*_z_*, *m_y_*, *A_x_*, *ω_d_*, *b_xy_*, *k_xy_*, *φ_s_*, and *K_s_*.

The following discusses the correlation between the bias, the scale factor, and the gyro quadrature error signal:

1. The parameter *φ_s_* is determined by the circuit. Any change in the phase value will have an effect on the gyroscope’s bias, scale factor, and quadrature error;
2. The parameter *K_s_* is determined by the circuit and the structure. Any change of this parameter will affect the gyroscope’s bias, scale factor, and quadrature error at the same time;
3. If the gyroscope fails after a strong impact or vibration, at this time, the parameters *m_y_*, *A_x_*, *ω_d_*, *b_xy__,_* and *k_xy_* will all change. The bias, scale factor, and quadrature signal amplitude will deviate from the initial value;
4. If the mass of the gyroscope changes slightly after strong impact or vibration, the gyroscope can still work. At this time, the parameter *m_y_* has changed. This will cause the structure’s modal stiffness *k_x_* and *k_y_*, coupling stiffness *k_xy_*, driving frequency *ω_d_*, and driving amplitude *A_x_* to change. The gyroscope’s bias, scale factor, and quadrature signal amplitude will all change accordingly;
5. If the damping of the gyroscope changes slightly after the impact or vibration, the damping coupling coefficient *b_xy_* will change slightly. The bias and quadrature signal amplitude will change accordingly;
6. If the stiffness of the gyroscope changes slightly after a strong impact or vibration, that is, *k_x_* and *k_y_* change, the coupling stiffness *k_xy_*, drive frequency *ω_d_*, and drive amplitude *A_x_* will change. The gyroscope’s bias, scale factor, and quadrature signal amplitude will all change accordingly.

In summary, any micro-mechanic change that causes the gyroscope bias and scale factor to change will cause the gyroscope quadrature error to change at the same time. The gyroscope quadrature error signal is closely related to the gyroscope bias and scale factor, and it changes synchronously in real time.

Therefore, by using the in-phase demodulation method to demodulate the quadrature error signal from the gyroscope sense modal displacement, and to detect whether the gyroscope quadrature error signal has changed, the health status of the gyroscope can be inferred in real time. If the gyroscope quadrature error signal significantly deviates from the original value, it can be concluded that the gyroscope state has changed significantly, and it can be concluded that the scale factor and bias of the gyroscope have changed significantly. The gyroscope has failed.

## 3. MEMS Gyroscope Design and Fabrication

**Figure 2:**
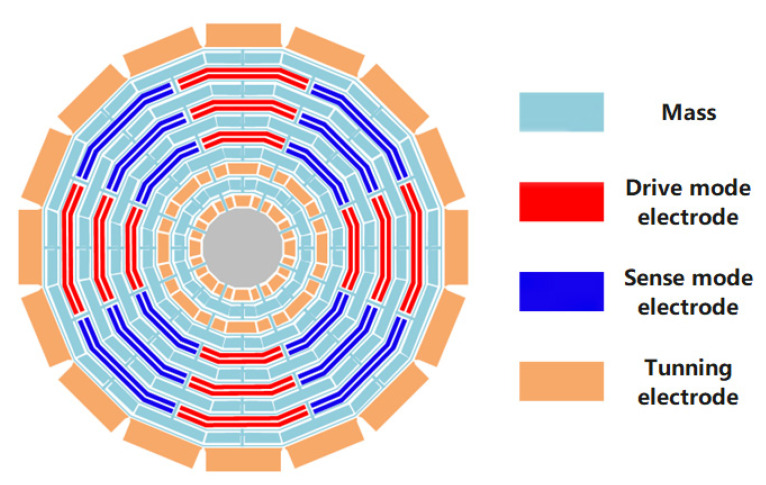
Schematic of a sixteen-sided cobweb-like MEMS gyroscope structure.

A sixteen-sided cobweb-like disk resonator MEMS gyroscope was designed and fabricated [26,27,28]. The gyroscope consisted of 10 concentric sixteen-sided cobweb-like rings connected through eight alternating spokes to a single central anchor, as shown in Figure 2. The diameter of the central anchor was 1.7 mm, and the thickness of each ring was 13 μm. The gyroscope drive modal excitation electrodes were distributed along the 0° and 180° angular distribution of structure, the drive modal detection electrodes were distributed along the 90° and 270°, the sense modal excitation electrodes were distributed along the 45° and 225°, and the sense modal detection electrodes were distributed along the 135° and 315°. All the excitation electrodes and the detection electrodes were nested in the middle of the multilayer sixteen-sided cobweb-like rings. The electrostatic tuning electrodes were evenly distributed on the outside and inside of the structure at 22.5° equal intervals.

**Figure 3:**
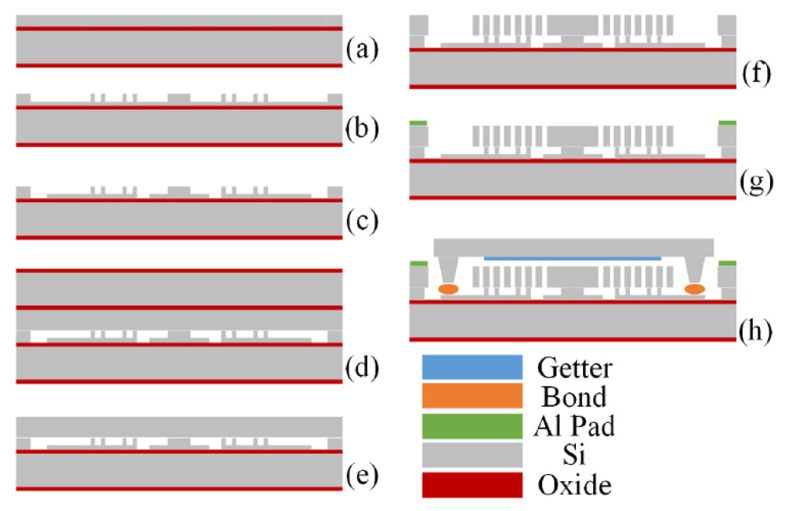
The fabrication process flow of the gyroscope. (**a**) a 6-inch SOI wafer with a 100 μm thick top device layer was used; (**b**) a cavity and anchors were etched; (**c**) the bottom electrodes layer was etched; (**d**) the device wafer was bonded; (**e**) the substrate of the handle wafer was removed; (**f**) the device structure was etch by DRIE; (**g**) the Al pads were fabricated; (**h**) the device was sealed by WLP process.

**Figure 4:**
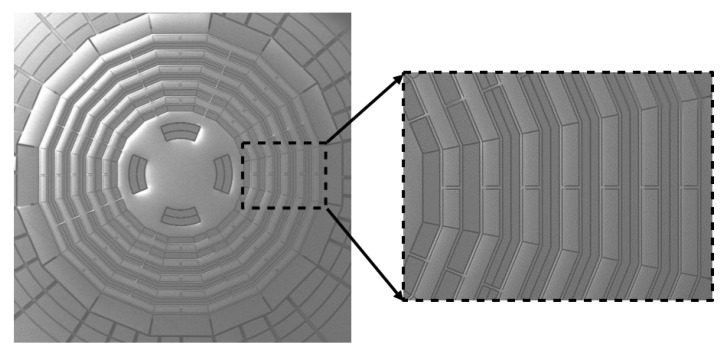
The SEM photo of the fabricated gyroscope.

The gyroscope was fabricated using SOI bulk processing, as shown in Figure 3. First, a 6-inch SOI wafer with a 100 μm thick top device layer was etched out of a cavity and anchor. Then the device wafer was fusion-bonded to the other SOI wafer, in which the active layer was patterned as bottom electrodes. The substrate of the device’s SOI wafer was then removed by deep reactive ion etching (DRIE), which left a 100 μm device layer on top of the bottom electrode wafer. Next, the cobweb-like disk resonator structure was formed in the device layer by DRIE. Finally, the released device wafer was hermetically sealed using a WLP (wafer-level package) process under a high-vacuum environment. An SEM photo of the fabricated gyroscope is shown in Figure 4.

## 4. BIST Design Based on Force-to-Balance Control Loop Architecture

**Figure 5:**
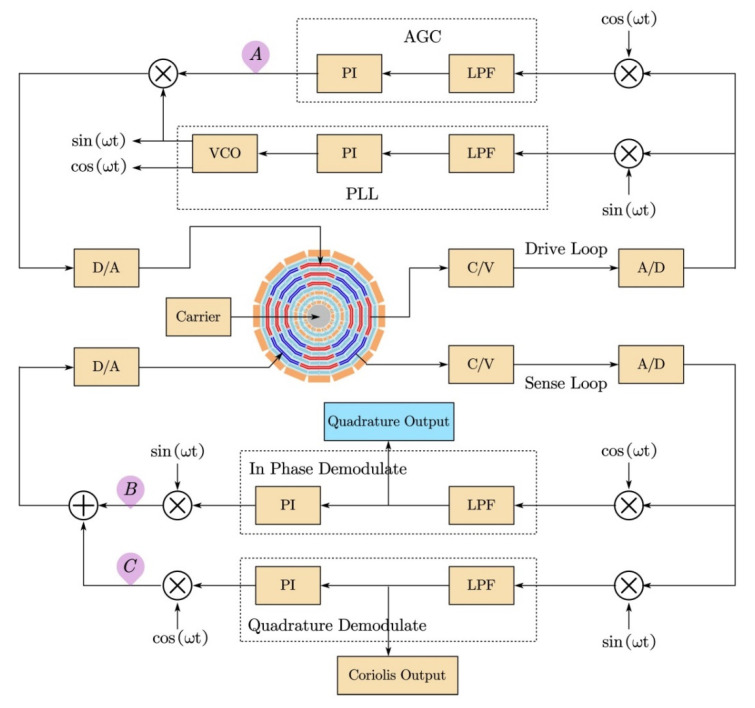
The diagram of the MEMS gyroscope circuit.

The block diagram of the gyroscope control system is shown in Figure 5. It contains a drive control loop and a force-to-balance control loop. The drive control loop maintains the frequency and amplitude of the drive mode through the phase-locked loop (PLL) and the automatic gain control (AGC). The sensitive control loop was achieved by the force-to-balance control method, and the in-phase and quadrature signal were PI controlled separately.

The in-phase signal, also known as the Coriolis signal, and the quadrature signal were decoupled using quadrature demodulation. The quadrature demodulation phase error had significant effects on our proposed self-test method. If there was a significant phase error, the Coriolis signal would be significantly coupled to the quadrature output. If the quadrature output signal varying with the input angular rate, the self-test method would fail. It is possible to minimize phase errors by using wide-bandwidth electronics and calibration methods as in [29].

Once the phase error was minimized, the mutual influence of the quadrature signal and Coriolis signal was suppressed. The quadrature output signal contained almost no Coriolis signal, but it was still a relatively constant signal which reflected the working condition of the MEMS structure and part of the sense loop. As shown in Figure 5, the quadrature output was directly affected by several blocks:

(a) The MEMS block: Both the Coriolis signal and quadrature signal were affected by the MEMS structure. If the MEMS structure malfunctioned, both the Coriolis signal and quadrature signal would change significantly. It was not likely that the Coriolis signal would change significantly, but the quadrature signal changed slightly;
(b) All of the drive loop blocks: any drive loop blocks’ malfunction would change both the Coriolis signal and quadrature signal significantly;
(c) The C/V, A/D, and D/A blocks of the sense loop: these circuit modules had no selectivity for the Coriolis signal and the quadrature signal, and the influence on the Coriolis signal and the quadrature signal was exactly the same;
(d) The Coriolis signal and quadrature signal control blocks, which contained the demodulators, lowpass filters, PI controllers, and modulators: Since the Coriolis signal and the quadrature signal were separated by quadrature demodulation under low-phase-error conditions, PI controllers for each signal were performed separately. Therefore, if the Coriolis signal control part failed, it would cause serious changes in the Coriolis signal but very weak changes in the quadrature signal. The self-test method that we proposed would be invalid. However, this is a very rare situation, as most MEMS sensor failures are due to the MEMS structure, not the circuit. We could design the circuit self-test function module to realize the detection of this part of the circuit, or redundantly design this function module to reduce the possibility of failure.

Above all, the MEMS gyroscope malfunctions during working conditions, especially after strong vibrations and shocks, are mostly due to the failure of the MEMS structure. The shock resistance technology for IC is relatively mature. The self-test method we proposed is the simple way to achieve the MEMS structure self-test.

## 5. BIST Simulation of the MEMS Gyroscope

**Figure 6:**
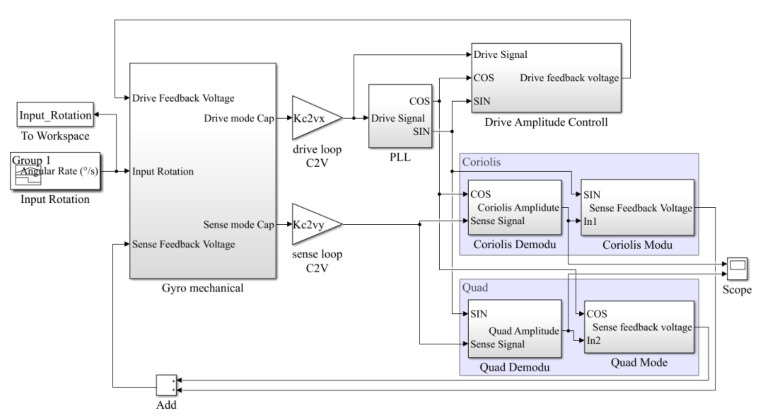
Gyroscope Simulink model.

The gyroscope Simulink simulation model was established as shown in Figure 6. The gyroscope drive loop used PLL (phase lock loop) for frequency control and used AGC (auto gain control) for amplitude control. The sense loop used the in-phase signal and the quadrature signal to demodulate the quadrature error signal and the Coriolis signal, respectively. Then each signal was PID adjusted separately. After quadrature modulation and in-phase modulation, they were summed and fed back to the sense excitation terminal. Thus, the drive closed-loop excitation and sensitive closed-loop detection were achieved.

In order to verify the gyroscope online BIST method, the gyroscope Simulink model was modified. We selected three points in the circuit and separately artificially set the values to zero. Meanwhile, we kept the other parts of the circuit the same to imitate the failure of the circuit. These three points were the AGC output at point A in the drive loop, the quadrature feedback control output at point B, and the Coriolis signal feedback control output at point C, as shown in Figure 5.

**Figure 7:**
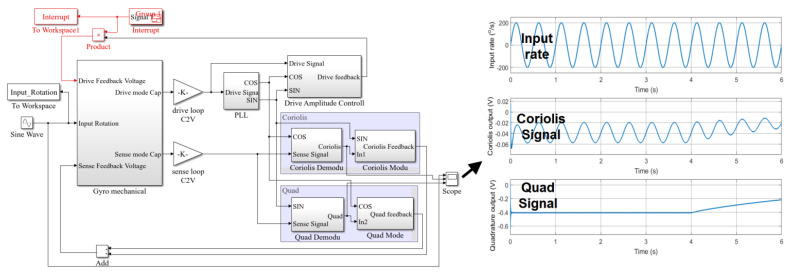
Simulink model for simulating the scenario A malfunction.

In scenario A, after the fourth second, the value of point A in the drive loop was modified to zero, which was used to simulate the failure phenomenon of the drive loop not working after the impact, as shown in Figure 7. It can be seen that after the fourth second, the amplitude of the gyroscope quadrature error shifted, and the Coriolis signal also shifted and its amplitude changed. Due to the gyroscope’s high-quality factor, the vibration of the gyroscope did not immediately attenuate to zero. Although the gyroscope still output a signal, the value was no longer correct, and the true input angular velocity could not be correctly calculated from the output signal of the gyro. The gyroscope failed.

**Figure 8:**
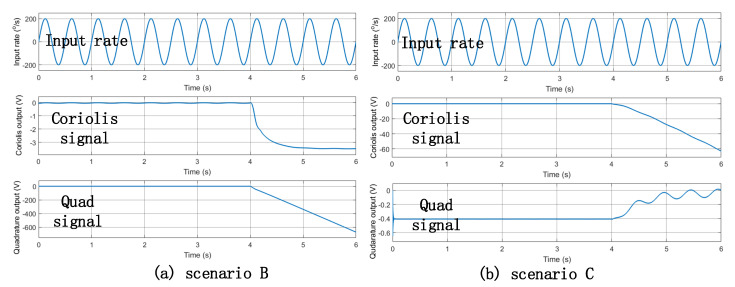
The simulation results under two malfunction scenarios respectively. (**a**) the simulation results under the scenario B malfunction; (**b**) the simulation results under the scenario C malfunction.

Similarly, in scenarios B and C, the value of point B and C were modified to zero, after the fourth second. The simulation results are shown in Figure 8. The Simulink model verified that, by monitoring whether the amplitude of the quadrature error signal deviated from the initial value, it was possible to determine whether the scale factor and bias of the gyroscope also deviated. When the amplitude of the quadrature error signal had a serious deviation, it could be concluded that the gyroscope failed.

## 6. The BIST Experiment

**Figure 9:**
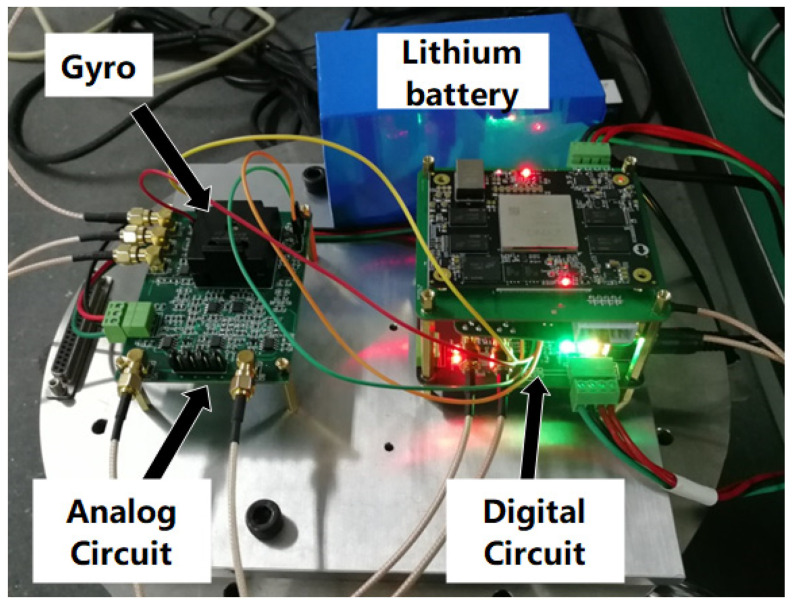
The photo of the gyroscope circuit.

The circuit was mainly composed of C/V conversion, front-end analog amplification, A/D conversion, FPGA, D/A conversion, and a carrier generator. Figure 9 is the photo of the gyroscope circuit. The sense modal displacement signal was input into the FPGA after C/V conversion and A/D sampling. Then, the quadrature demodulation and the in-phase demodulation were performed, respectively, in the FPGA. The amplitude of the quadrature signal demodulation was output synchronously with the Coriolis signal.

Due to the phase shift error of the gyroscope circuit, there was a quadrature error signal in the gyroscope output Coriolis signal obtained by quadrature demodulation, and there was also a Coriolis signal in the gyroscope output quadrature error signal. Therefore, in the actual circuit, both the quadrature demodulation reference signal and in-phase demodulation reference signal were slightly phase-shifted.

We chose two gyroscopes to carry out the experiment. One gyroscope was used for the electrical test, which imitated the circuit malfunction scenarios A, B, and C. The other gyroscope was used for the mechanical shock test.

### 6.1. The Electric Malfunction Imitated Test

**Figure 10:**
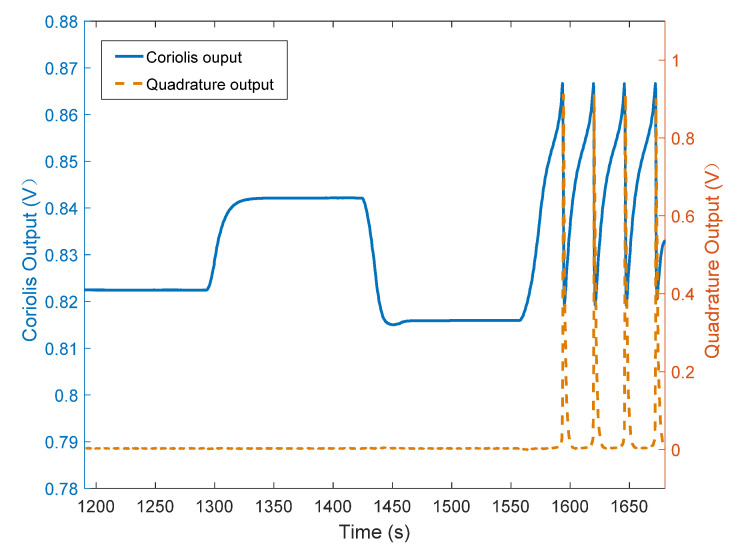
The Coriolis output signal becoming unstable in the Scenario C imitated experiment.

Three MEMS gyroscope circuit malfunction scenarios were imitated, and the gyroscope was tested on a turntable. The scale factor, bias, and the amplitude of quadrature error were calculated. Table 1 shows the experimental results. In scenarios A and B, the Coriolis output signal did not have a linear relationship with the input angular velocity. The details are as follows:

1. Scenario A: The value of point A was set to zero. Once the AGC output of the drive loop was zero, the gyroscope structure did no resonate. The drive loop of the gyroscope malfunctioned. The output value of the Coriolis signal and the quadrature signal were almost zero, which shifted from the correct ones;
2. Scenario B: The value of point B was set to zero. In this scenario, the quadrature control loop changed from a closed loop to an open loop. Due to the small phase error, the quadrature feedback signal would not be coupled to the Coriolis signal through the demodulation signal process. Ideally, the Coriolis signal would not change. However, in the real situation, because the quadrature feedback signal was zero, the structure’s quadrature error was not effectively suppressed. The original closed loop of the Coriolis signal became unstable. The true Coriolis signal was subsumed in the error signal. The gyroscope output had no linear relationship with the input angular velocity;
3. Scenario C: The value of point C was set to zero. In this scenario, the Coriolis control loop changed from a closed loop to an open loop. Due to the small phase error, the Coriolis feedback signal would not be coupled to the quadrature signal through the demodulation signal process. The gyroscope output had a linear relationship with the input angular velocity, but the gyroscope bias and the output of the quadrature signal shifted from the normal ones. During this experiment, it was found that the Coriolis output signal became unstable when input angular velocity reached −100°/s, as shown in Figure 10.

The electrical malfunction imitated test results show that most of gyroscope circuit failures could be detected by monitoring the quadrature error signal.

### 6.2. The Mechanical Shock Test

**Figure 11:**
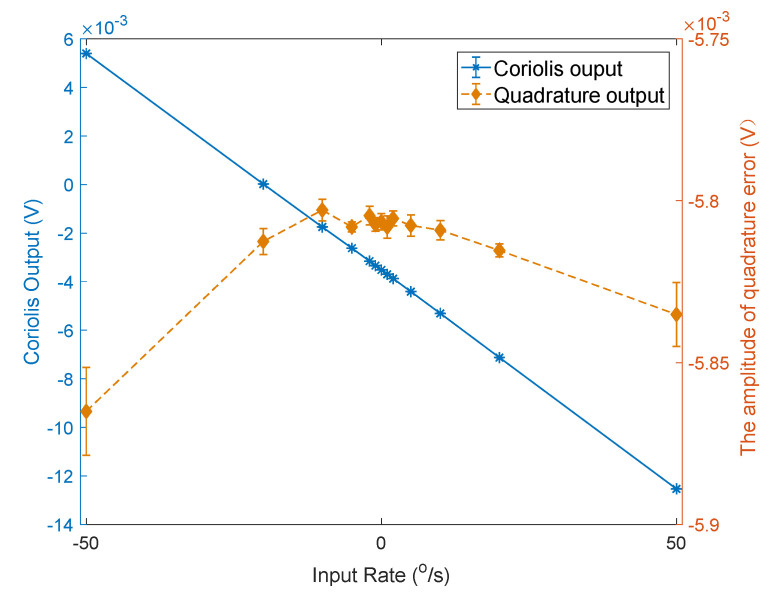
The variation curves of the Coriolis signal and the amplitude of the quadrature error signal with the input angular velocity.

Figure 11 shows the variation curves of the Coriolis signal and the amplitude of the quadrature error signal of one of the test gyroscopes as the input angular velocity was changed. It can be seen that the amplitude of the quadrature error signal hardly changed with alterations in the input angular velocity.

**Figure 12:**
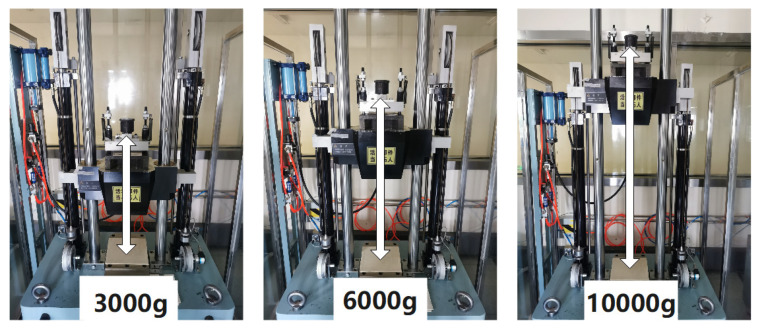
The photo of gyroscope impact tests.

In order to eliminate the experimental error caused by the impact of the PCB, only the MEMS gyroscope was mounted on the mechanical impact table to perform impacts of 3000 g, 6000 g, and 10,000 g along the gyroscope input axis, as shown in Figure 12.

After each impact, the MEMS gyroscope was retested on a turntable. The scale factor, bias, and the amplitude of quadrature error were calculated.

**Figure 13:**
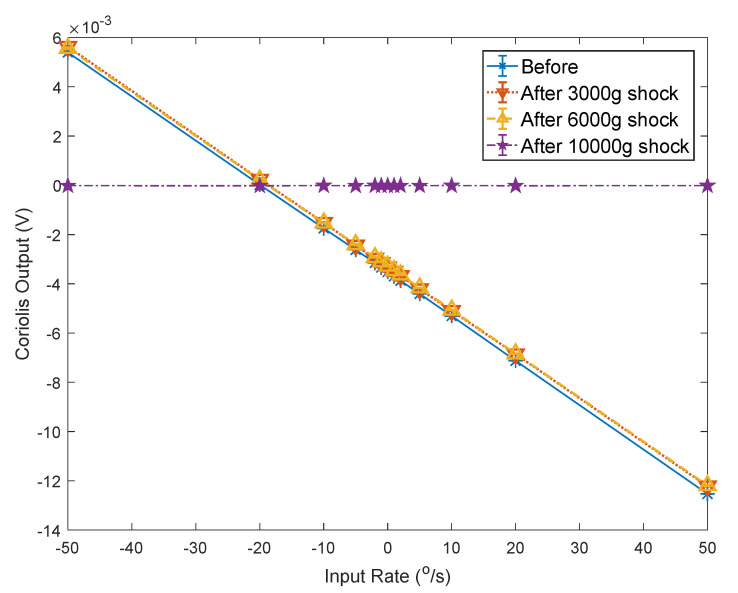
The experimental curves of scale fact before and after the impacts.

**Figure 14:**
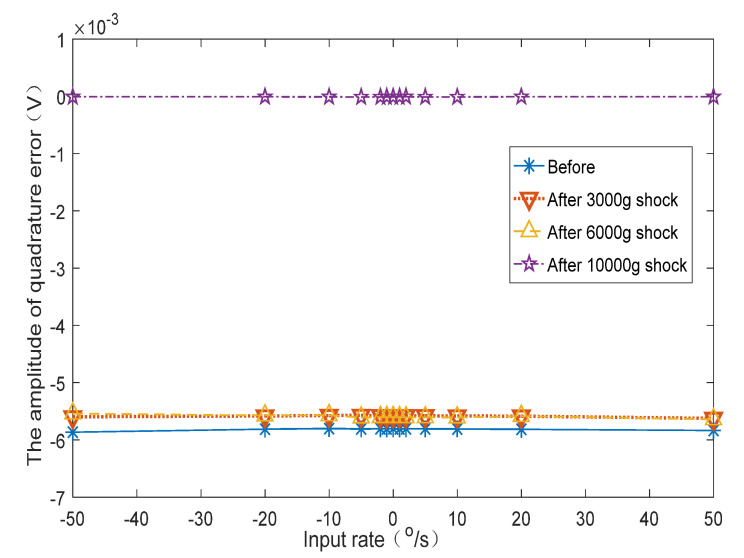
The experimental curves of the quadrature error amplitude before and after the impacts.

Figure 13 shows the curves of the variation of the gyroscope’s Coriolis signal with changes in the input angular velocity before and after impact. Figure 14 shows the variation curve of quadrature error amplitude with changes in the input angular velocity before and after impact. Table 2 shows the calculated average value of the scale factor, bias, and quadrature error amplitude before and after the impact. Table 3 shows the variation of scale factor, bias, and quadrature error amplitude after the impacts compared to the original value.

The test results show that after 3000 g and 6000 g both the scale factor and bias changed. The amplitude of the quadrature error also deviated from the original value.

After the impact of 10,000 g, the gyroscope failed and the output Coriolis signal was incorrect. At this time, the amplitude of the quadrature error changed −99.82% compared to the value before the impact. The amplitude of the quadrature error significantly deviated from the original value.

Therefore, by monitoring the change in the quadrature error amplitude in the working state, it can be inferred whether the gyroscope scale factor and bias have changed significantly, and it can also be concluded whether the gyroscope output Coriolis signal is reliable.

## 7. Discussion

In order to perform BIST of the MEMS gyroscope in the working state and infer whether the gyroscope output signal was reliable, we proposed monitoring the quadrature error signal to achieve online BIST for MEMS gyroscope under a low-phase-error demodulation condition. The gyroscope Simulink model was established to verify the proposed BIST method. The structure and circuit of the sixteen-sided cobweb-like MEMS gyroscope were designed and fabricated. The circuit output the Coriolis signal and the quadrature error amplitude signal synchronously. Both electric malfunction imitated tests and mechanical shock tests were developed. The 3000 g, 6000 g, and 10,000 g impact tests were carried out. The gyroscope Coriolis signal and the quadrature error amplitude signal were measured on the turntable before and after the impact.

After the 6000 g impact, the quadrature error amplitude signal changed by −3.74%. At this time, the scale factor changed by −1.02%, and the bias changed by −5.76%. The gyroscope failed after the 10,000 g impact, and the gyroscope quadrature error amplitude signal changed by −99.82%. Therefore, by monitoring the change in the quadrature error amplitude, it could be inferred whether the gyroscope scale factor and bias changed significantly. The BIST method based on quadrature error can be used for MEMS gyroscope online self-test without interfering with the gyroscope output.

Future works will focus on improving this self-test method, especially having a relatively large demodulation phase error.

## 8. Patents

A Chinese invention patent application has been submitted (Rui Feng. A micro-mechanical gyroscope fault self-detection method based on quadrature error signal. Application No. 201710182048.2).

## Figures and Tables

**Table 1:** The experimental results of the electric malfunction imitated test.

	Normal Condition	Scenario A	Scenario B	Scenario C
Scale factor (V/°/s)	−1.73 × 10^−4^	-	-	−2.11 × 10^−4^
Bias (V)	−5.12 × 10^−4^	−4.05 × 10^−7^	−1.19	3.33 × 10^−2^
Bias stability (V)	1.29 × 10^−5^	1.33 × 10^−5^	1.13	2.51 × 10^−4^
The average of the quadrature signal (V)	2.92 × 10^−3^	4.58 × 10^−7^	8.84 × 10^−3^	2.54 × 10^−3^
The standard deviation of the quadrature signal (V)	2.24 × 10^−6^	1.13 × 10^−6^	1.81 × 10^−2^	5.19 × 10^−5^

**Table 2:** The experimental results of scale factor, bias, and quadrature error amplitude before and after the impacts.

	Scale Factor (V/°/s)	Bias (°/s)	The Amplitude of Quadrature Error (V)
before	−1.79 × 10^−4^	19.70	−5.81 × 10^−3^
after 3000 g	−1.78 × 10^−4^	18.59	−5.58 × 10^−3^
after 6000 g	−1.77 × 10^−4^	18.56	−5.59 × 10^−3^
after 10,000 g	—	−1093.17	−1.02 × 10^−5^

**Table 3:** The variation of scale factor, bias, and quadrature error amplitude after the impacts compared to the original value.

	Scale Factor Variation	Bias Variation	Quadrature Error Amplitude Variation
after 3000 g	−0.49%	−5.62%	−3.94%
after 6000 g	−1.02%	−5.76%	−3.74%
after 10,000 g	−100%	−5648.09%	−99.82%

## Data Availability

The data presented in this study are available from the corresponding author, [R.F.], upon reasonable request.
